# Collision metastasis of lung adenocarcinoma and melanoma presenting as a fungating axillary mass

**DOI:** 10.1016/j.jdcr.2026.05.029

**Published:** 2026-05-20

**Authors:** Malak Husseinali, Erica Ogwumike, Rishabh Lohray, Nisha Ramani, Ida Orengo, Theodore Rosen

**Affiliations:** a School of Medicine, Baylor College of Medicine, Houston, Texas; b Department of Dermatology, Baylor College of Medicine, Houston, Texas; c Department of Pathology, Baylor College of Medicine, Houston, Texas; d Chief of Dermatology, Michael E. DeBakey VA Medical Center, Houston, Texas

**Keywords:** adenocarcinoma, adenocarcinoma of lung, collision tumors, immunohistochemistry, lung neoplasms, lymph nodes, melanoma, neoplasm metastasis

## Introduction

Collision metastasis is a rare pathologic phenomenon in which 2 histologically distinct malignancies converge within a single metastatic site. Although most reported cases are identified incidentally, recognition carries important diagnostic and therapeutic implications, particularly in patients with advanced disease. We report a case of collision metastasis involving lung adenocarcinoma and melanoma presenting as a rapidly enlarging, fungating axillary mass, an unusual clinical presentation that underscores the importance of careful histopathologic evaluation in atypical cutaneous and nodal lesions.

## Case report

A 77-year-old White male with a history of hypertension and hyperlipidemia presented with a rapidly enlarging, fungating right axillary mass. He had no personal history of malignancy, known immunosuppression, or history of tobacco use. He resided primarily in Mexico and endorsed significant sun exposure, including approximately 10 years of agricultural work and prior residence in Miami, Florida. Family history was notable for a sister with nonmelanoma skin cancers.

**Fig 1:**
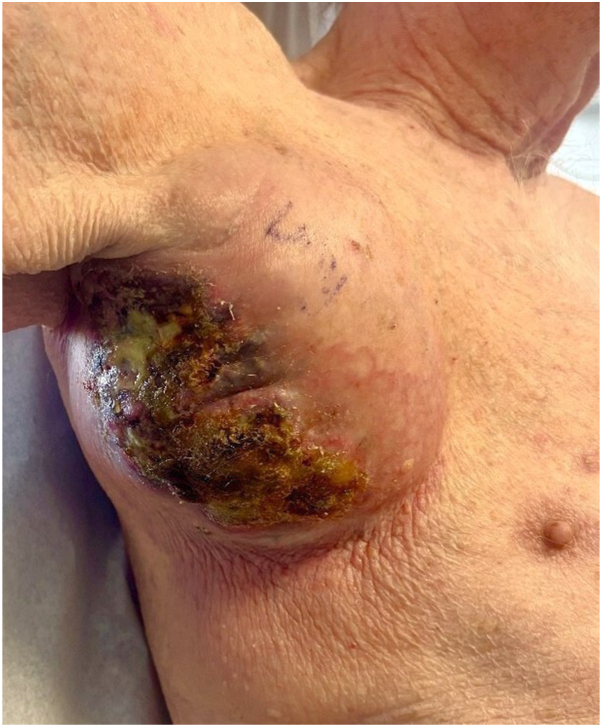
Collision metastasis of lung adenocarcinoma and malignant melanoma. Clinical photograph showing a fungating, ulcerated right axillary mass measuring approximately 10 cm.

Approximately 2 months prior to presentation, the patient noticed a painless, “peanut-sized” mass in the right axilla. Over the ensuing weeks, the lesion rapidly increased in size, became painful, and developed overlying erythema, ulceration, and serosanguinous drainage. He additionally reported progressive fatigue, decreased appetite, and unintentional weight loss of 5-6 pounds without fevers, chills, or night sweats. Physical examination revealed a fungating, ulcerated subcutaneous mass measuring 10 cm in greatest dimension in the right axilla, with surrounding induration and drainage (Fig 1). Multiple firm subcutaneous nodules were palpated in the left axilla; no cervical lymphadenopathy was present. On full body skin exam, the patient had multiple nonmelanoma skin cancers but no outward signs of melanoma. A single skin biopsy was done in the most suspect area overlying the mass, and no evidence of melanoma was found.

Initial imaging with computed tomography of the chest, abdomen, and pelvis demonstrated bulky right greater than left axillary lymphadenopathy, with the largest right axillary node measuring up to 8.5 cm. A 4.2-cm mass was identified in the left lower lobe of the lung, accompanied by multiple smaller bilateral pulmonary nodules. Additional findings included a 5.2-cm hypodense splenic mass, several peritoneal implants, and soft tissue nodules adjacent to the kidneys, raising concern for disseminated metastatic disease. Magnetic resonance imaging of the brain revealed 3 subcentimeter enhancing lesions within the left cerebral hemisphere suspicious for intracranial metastases.

**Fig 2:**
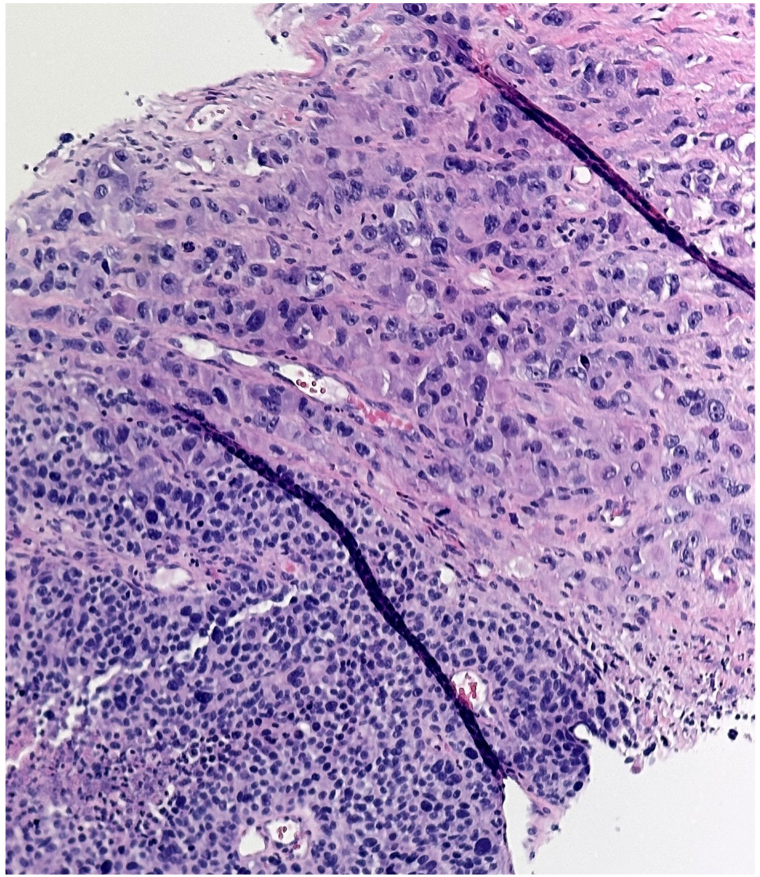
Histopathologic features of collision metastasis. Hematoxylin and eosin–stained section of the right axillary lymph node showing 2 morphologically distinct malignant populations in close proximity (×10).

**Fig 3:**
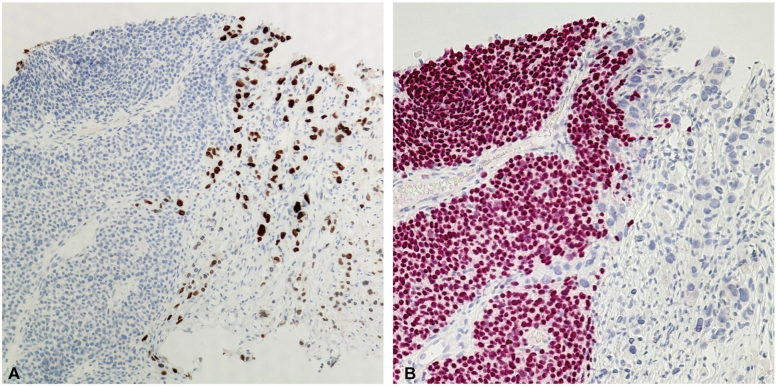
Immunohistochemical confirmation of collision metastasis. **A,** TTF-1 highlights the metastatic lung adenocarcinoma component (×20). **B,** SOX10 shows nuclear positivity in the melanoma component (×10).

Ultrasound-guided core needle biopsies of the right axillary mass were performed by interventional radiology. The core biopsy demonstrated nodal architecture, confirming that the melanoma represented nodal metastasis. Histopathologic examination demonstrated 2 morphologically and immunophenotypically distinct malignant populations in close proximity, consistent with a collision metastasis (Fig 2). One tumor population consisted of large pleomorphic epithelioid cells with prominent nucleoli. These cells showed strong positivity for thyroid transcription factor-1 (TTF-1) and CK7, supporting a diagnosis of metastatic lung adenocarcinoma (Fig 3, *A*). The second population was composed of smaller malignant cells with less pleomorphism, which stained positively for several melanoma-associated markers SOX10, HMB45, and Melan-A, and was negative for TTF-1 and CK7, consistent with metastatic melanoma (Fig 3, *B*). Immunohistochemical stains for chromogranin, synaptophysin, CD56, PAX8, and CK20 were negative in both sets of cells. The presence of 2 distinct malignant clones with non-overlapping immunoprofiles confirmed the diagnosis of collision metastasis of lung adenocarcinoma and melanoma involving the axillary lymph node. Given the advanced histologic findings, the patient declined further surgical intervention and additional tissue sampling.

Following diagnosis, the patient was evaluated by medical and radiation oncology and planned for palliative radiation therapy to the bilateral axillae for local disease control and focused radiation to intracranial metastases.

## Discussion

Collision metastasis refers to the coexistence of 2 histologically distinct malignancies within a single metastatic site. True collision metastases are rare and are most often identified incidentally during histopathologic evaluation of lymph nodes or visceral lesions.^1^

Within the broader literature on collision tumors and tumor-to-tumor metastasis, lung carcinoma is among the most frequently reported metastatic donor tumors, a pattern attributed to its high incidence and aggressive metastatic behavior.^1^^,^^2^ Lung cancer–associated collision tumors commonly involve hematologic malignancies, particularly lymphoma, whereas collisions with other solid tumors are heterogeneous and largely limited to isolated case reports.^3^, ^4^, ^5^

Melanoma-associated collision metastases are uncommon and are most often identified in lymph nodes during staging or assessment of disease progression. Reported collision partners include hematologic malignancies and solid tumors of the bladder, colon, breast, and endocrine organs.^6^ Melanoma also demonstrates a notable capacity for tumor-to-tumor metastasis, with reported involvement of preexisting intracranial, endocrine, and visceral neoplasms.^2^^,^^6^^,^^7^ In contrast, the majority of melanoma-associated collision tumors involve cutaneous–cutaneous neoplasms, such as collisions with basal cell or squamous cell carcinoma.^7^ These cutaneous collisions are relatively common and typically lack systemic prognostic or therapeutic significance, whereas collision metastases involving melanoma and a second internal malignancy remain rare and clinically consequential.

Collision metastasis specifically involving lung carcinoma and melanoma appears exceptionally rare, with only a limited number of cases reported.^6^^,^^8^, ^9^, ^10^ Described sites include the central nervous system, lymph nodes, and serosal or cutaneous metastatic deposits. Diagnosis has relied on careful histopathologic evaluation supported by immunohistochemistry to distinguish melanocytic from epithelial tumor populations.^8^^,^^9^ Available data from melanoma-associated collision tumors suggest that prognosis is largely driven by the biologically more aggressive or advanced malignant component, although follow-up remains limited.^6^^,^^8^, ^9^, ^10^

Several mechanisms have been proposed to explain collision metastases, though the process is likely multifactorial. Suggested explanations include chance apposition, shared lymphatic or vascular drainage pathways, and permissive tumor microenvironments consistent with “seed and soil” dynamics, in which local inflammatory or stromal factors facilitate secondary tumor seeding.^1^^,^^2^ In the absence of a unifying biologic mechanism, some collision metastases may represent coincidental convergence of independent metastatic processes rather than a defined tumor-tumor interaction.^1^^,^^5^ This distinction is clinically relevant, as collision metastases may be underrecognized or misclassified as intratumoral heterogeneity or dedifferentiation, particularly within limited biopsy specimens.

From a diagnostic standpoint, recognition of collision metastasis requires adequate tissue sampling and comprehensive immunohistochemical analysis.^7^ Therapeutically, accurate classification is essential, as management must account for 2 biologically distinct malignancies with potentially divergent prognostic and treatment implications.^1^ Reported management strategies have included combinations of systemic therapy, local therapies such as surgery or radiation for symptomatic control, and immunotherapy or targeted therapy. Outcomes remain quite variable, with data limited to case reports.^1^^,^^8^^,^^10^ In the present case, identification of concurrent metastatic melanoma and lung adenocarcinoma within a fungating axillary mass informed multidisciplinary decision-making and supported a palliative, symptom-directed treatment approach.

## Conflicts of interest

None disclosed.
